# Transcriptome Analysis by RNA–Seq Reveals Genes Related to Plant Height in Two Sets of Parent-hybrid Combinations in Easter lily (*Lilium longiflorum)*

**DOI:** 10.1038/s41598-020-65909-x

**Published:** 2020-06-03

**Authors:** Jewel Howlader, Arif Hasan Khan Robin, Sathishkumar Natarajan, Manosh Kumar Biswas, Kanij Rukshana Sumi, Cheon Young Song, Jong–In Park, Ill–Sup Nou

**Affiliations:** 1 Department of Horticulture, Sunchon National University, 255, Jungang–ro, Suncheon, Jeonnam 57922 Republic of Korea; 2 Department of Horticulture, Patuakhali Science and Technology University, Dumki, Patuakhali 8602 Bangladesh; 3 Department of Genetics and Plant Breeding, Bangladesh Agricultural University, Mymensingh, 2202 Bangladesh; 4 Department of Fisheries Science, Chonnam National University, 50, Daehak–ro, Yeosu, Jeonnam 59626 Republic of Korea; 5 Department of Aquaculture, Patuakhali Science and Technology University, Dumki, Patuakhali 8602 Bangladesh; 6 Department of Floriculture, Korea National College of Agriculture and Fisheries, 1515, Kongjwipatjwi–ro, Wansan–gu, Jeonju–si, Jeollabuk–do 54874 Republic of Korea

**Keywords:** Biotechnology, Genetics, Plant sciences

## Abstract

In this study, two different hybrids of Easter lily (*Lilium longiflorum)*, obtained from two cross combinations, along with their four parents were sequenced by high–throughput RNA–sequencing (RNA–Seq) to find out differentially expressed gene in parent-hybrid combinations. The leaf mRNA profiles of two hybrids and their four parents were RNA–sequenced with a view to identify the potential candidate genes related to plant height heterosis. In both cross combinations, based to morphological traits mid–parent heterosis (MPH) was higher than high–parent heterosis (HPH) for plant height, leaf length, and number of flowers whereas HPH was higher than MPH for flowering time. A total of 4,327 differentially expressed genes (DEGs) were identified through RNA–Seq between the hybrids and their parents based on fold changes (FC) ≥ 2 for up– and ≤ –2 for down–regulation. Venn diagram analysis revealed that there were 703 common DEGs in two hybrid combinations, those were either up– or down–regulated. Most of the commonly expressed DEGs exhibited higher non–additive effects especially overdominance (75.9%) rather than additive (19.4%) and dominance (4.76%) effects. Among the 384 functionally annotated DEGs identified through Blast2GO tool, 12 DEGs were up–regulated and 16 of them were down–regulated in a similar fashion in both hybrids as revealed by heat map analysis. These 28 universally expressed DEGs were found to encode different types of proteins and enzymes those might regulate heterosis by modulating growth, development and stress–related functions in lily. In addition, gene ontology (GO) analysis of 260 annotated DEGs revealed that biological process might play dominant role in heterotic expression. In this first report of transcriptome sequencing in Easter lily, the notable universally up-regulated DEGs annotated ABC transporter A family member–like, B3 domain–containing, disease resistance RPP13/1, auxin–responsive SAUR68–like, and vicilin–like antimicrobial peptides 2–2 proteins those were perhaps associated with plant height heterosis. The genes expressed universally due to their overdominace function perhaps influenced MPH for greater plant height― largely by modulating biological processes involved therein. The genes identified in this study might be exploited in heterosis breeding for plant height of *L. longiflorum*.

## Introduction

Heterosis refers to the higher performance in any trait (s) of interest of F_1_ hybrid as compared to homozygous parental lines. Charles Darwin^1^ described this phenomenon for the first time ever and later Shull^2^ and East^3^ independently confirmed this phenomenon. Improvements in many traits of different crop and livestock species have been achieved by exploiting heterosis during the last centuries^4–8^. Numerous investigations attempted generalizing the molecular basis of heterosis^9^. Despite, a conclusive molecular mechanism of heterosis remains elusive^10^.

Heterosis could be either positive or negative depending on traits of breeding interest. For example, the yield is a positive heterotic trait and earliness is a negative heterotic trait^11–13^. There are two classic hypotheses namely dominance hypothesis and overdominance hypothesis those are used to differentiate allelic variations in expression in between hybrids and inbred parents^14^. In dominance hypothesis, alleles related to the desired trait of interest from the one parent suppress the alleles linked to less desired trait of interest from another parent. In contrast, overdominance heterosis occurs due to the simultaneous action of two parental alleles resulting different superior traits in a hybrid. These two hypotheses are redefined in relation to additivity and non–additivity to classify differentially expressed genes (DEGs) between parent and hybrids^15,16^. In additive gene effect, the expression level of DEGs in hybrid is similar to the average of both parents (mid–parental expression) while in non–additive effect, the expression level is different from the parental mean. Non–additive gene effect has also been classified into high parent dominance (high parent–like expression), low parent dominance (low parent–like expression), overdominance (above high parent expression value) and underdominance (below low parent expression value)^15^. Experimental evidences show that heterosis in most cases manifested by the incidence of non–additive gene expression^17–22^. In a few cases, however, this phenomenon was also reported to be arisen from additive gene expression levels^20,22,23^.

Next–generation sequencing (NGS) especially high–throughput RNA sequencing (RNA–seq) technology is advantageously used for discovering heterotic genes over other expression profiling technologies in plants and animals^13,24–26^. In plants, using RNA–Seq technology, expression patterns of DEGs between hybrids and their parents can be analyzed that might contribute to understanding the genetic basis of heterosis^13,27,28^. Transcriptome analyses revealed that in nascent hexaploid wheat dominance gene expression was predominant for allopolyploid heterosis^15^ but in tobacco, overdominance was key factor for nicotine biosynthesis^29^. Dominance and overdominance effects were displayed by heterotic genes associated with the development of ears earlier in maize inflorescence^30^. In chrysanthemum, two flowering traits— initial blooming time and duration of flowering are regulated by the presence of two pairs of major genes where additivity is predominant^31^. By contrast, the non–additivity might be related to early vegetative development, increased photosynthesis, cell size and number in hybrids that might play the key roles of contribution to the biomass heterosis in Arabidopsis^21,32^.

Heterosis in a hybrid as estimated by vegetative growth, flowering time, yield and resistance to biotic and abiotic stresses was shown be regulated by differential gene expression patterns in plants^4^. In contrast to self–fertilized plants, cross–fertilized one exhibits 10–200% higher heterosis in wheat and maize. Interestingly, when two genetically diverse alleles meet and attain a heterozygous state, the resultant genotypes experience a period of genomic turbulence a so called ‘*Genomic Shock*’ until a stable homeostasis is established^33,34^. This genomic turbulence might trigger the wide range of regulatory genes with differential gene expression patterns^35^. Heterozygosity of the regulating genomic regions that brings about superiority in hybrids in case of most of the quantitative traits could be either monogenic or polygenic^36^. For example, superior hybrid performance in relation to several agronomic traits in tomato^37^ and lily^38^ might be resulted from a single heterozygous gene linked to heterosis. In contrast, performance of most economically interesting traits of maize^39^, Arabidopsis^40^ and rapeseed^41^ might be regulated by multiple heterotic genes.

Lilies (*Lilium* spp., 2n = 24), the bulbous monocots, are the outcrossing perennial herbs of the Liliaceae family. Lilies have high global market due to their incomparable beauty and commercial value^42,43^. Some Asian countries including China, Korea, Japan and Nepal are considered as one of the important center of diversity of lilies^44^. Lily is a highly popular cut flower in Korea. Annual production of lilies in Korea has an economic value of about US$ 34 million^45,46^. In lily, modern hybrid breeding exploit hybrid vigor of inter-sectional hybridization of Longiflorum, Oriental and Asiatic hybrids^47^. Breeders selects those hybrids for plant height; number of leaves, flowers and bulbils; leaf area; flower size; days to flowering and petal area which indicated that those traits were often found heterotic^48–50^.

Despite the economic and aesthetic importance of lilies, no attempt was made to date that investigate the heterotic genes related to any phenotypic traits in *Lilium* spp. Besides, RNA–Seq facility may help identify heterotic genes to understand types of proteins or functional domains of the genes are involved in heterosis. In this study, we investigated the heterosis for plant height in Easter lily (*Lilium longiflorum*) by performing RNA–Seq analyses in two hybrids with their respective four parental inbred lines. The objectives of this study were to identify heterosis related DEGs involved in plant height and to interpret underlying genetic reasons that control heterosis in intra-specific hybrids in *L. longiflorum*. We predicted that DEGs in hybrids compared to parents in relation to these traits would exhibit heterosis for plant height in *L. longiflorum*. In this study, we identified some commonly expressed up– and down–regulated candidates DEGs in hybrids in relation to their respective parental lines and analyzed their expression patterns. We then explored the functional features of the candidate genes to understand the molecular mechanism and genetic reason of heterosis for plant height in *L. longiflorum*.

## Results

### Characterization of Hybrids and their parental lines

**Figure 1:**
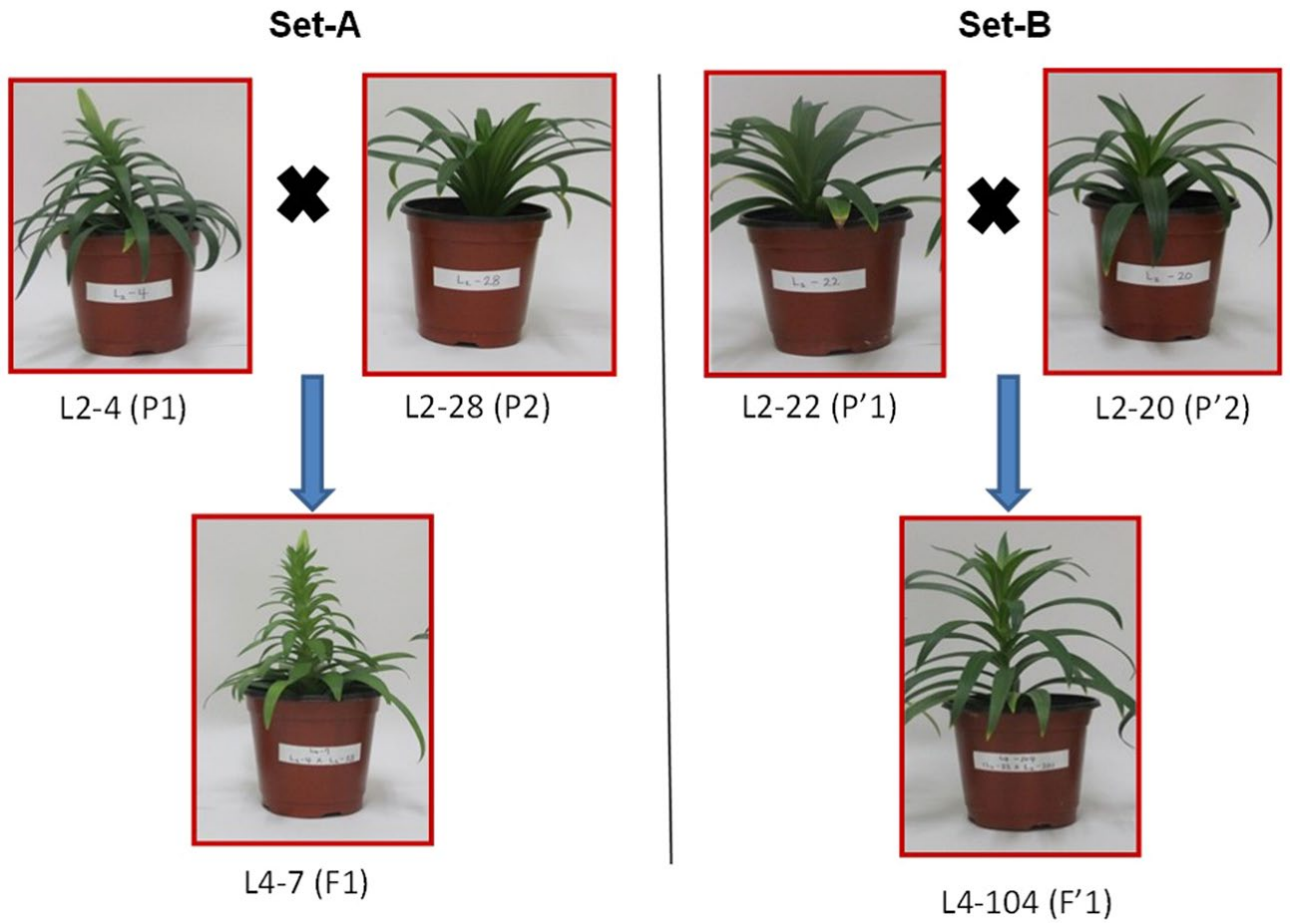
Comparisons of phenotypic variations in hybrids with their respective parents of *L*. *longiflorum*. The left panel (Set–A) shows the hybrid (F1, L4–7) and its parents of L2–4 (P1) and L2–28 (P2) while the right panel (Set–B) shows the hybrid (F′1, L4–104) and its parents of L2–22 (P′1) and L2–20 (P′2) at 4 months seedling stage.

We used two different intra–species crosses (L4–7, L2–4 × L2–28 and L4–104, L2–22 × L2–20) of *Lilium longiflorum* (Easter lily) to investigate the phenotypic variations between hybrids and their respective inbred parents (Fig. 1).

Plant height, leaf length, flowering time, number of flowers, and flower diameter showed significant (*p* < 0.05) phenotypic variation between hybrids and their inbred parents in both cross combinations (Table 1). The plant height in hybrid L4–7 (47.1 cm) was 21.0% and 23.67% higher than the high–parent or better–parent (37.2 cm) and mid–parent (35.95 cm), respectively, whereas that was 23.3% and 32.33% higher than the high–parent (39.5 cm) and mid–parent (35.05 cm), respectively in hybrid L4–104 (51.8 cm) (Table 1). The leaf length in hybrid L4–7 (14.5 cm) was 6.89% lower than the high–parent (15.5 cm) and 4.13% higher than the mid–parent (13.9 cm) whereas that in hybrid L4–104 (18.5 cm) was 29.7% and 31.8% higher than the high–parent (13.0 cm) and mid–parent (12.6 cm), respectively (Table 1). The days to flowering in hybrid L4–7 (184 days) was 2.1% and 3.53% lower than the high–parent (188 days) and mid–parent (190.5 days), respectively, whereas that in hybrid L4–104 (183 days) was 3.27% and 4.64% lower than the high–parent (189 days) and mid–parent (191.5 days), respectively (Table 1). The number of flowers in hybrid L4–7 (10.3) was 44.6% and 48.5% higher than the high–parent (5.7) and mid–parent (5.3), respectively, whereas that in hybrid L4–104 (7.5) was 49.3% and 50.0% higher than the high–parent (3.8) and mid–parent (3.75), respectively (Table 1). The flower diameter in hybrid L4–7 (13.3) was 8.2% and 6.7% lower than the high–parent (14.4) and mid–parent (14.2), respectively, whereas that in hybrid L4–104 (13.8) was 3.6% and 1.4% lower than the high–parent (14.3) and mid–parent (14.0), respectively (Table 1).

**Table 1:** Mid–parent heterosis (MPH) and high–parent heterosis (HPH) of different phenotypic traits in six genotypes of *L. longiflorum*. MPH and HPH were calculated using the following formulas, MPH = (F1 − MP)/MP in % and HPH = (F1 − HP)/HP in %, where F1 denotes the average performance of the hybrid, MP denotes the average performance of the two parents, and HP denotes the average performance of the better–parent between two parents. Each data represent average of three plants.

Phenotypic traits	Cross combination A					Cross combination B				
	L2–4 (P1) (♀)	L4–7 (F1)	L2–28 (P2) (♂)	MPH (%)	HPH (%)	L2–22 (P′1) (♀)	L4–104 (F′1)	L2–20 (P′2) (♂)	MPH (%)	HPH (%)
Plant height (cm)	37.2^b^ ± 2.4	47.1^a^ ± 3.4	34.7^c^ ± 4.2	31.01^**^	26.61^**^	39.5^b^ ± 3.7	51.8^a^ ± 5.9	30.6^c^ ± 4.3	47.79^**^	31.14^**^
Leaf length (cm)	12.3^c^ ± 1.8	14.5^b^ ± 1.2	15.5^a^ ± 2.4	4.32^**^	-6.45^**^	13.0^b^ ± 1.7	18.5^a^ ± 2.1	12.2^c^ ± 1.8	46.83^**^	42.31^**^
Days to flowering	193^a^	184^c^	188^b^	-3.41^**^	-4.66^**^	194^a^	183^c^	189^b^	-4.44^**^	-5.67^**^
Number of flowers	4.9^c^ ± 1.1	10.3^a^ ± 2.1	5.7^b^ ± 1.3	94.34^**^	80.70^**^	3.7^bc^ ± 1.7	7.5^a^ ± 2.1	3.8^bc^ ± 1.2	100^**^	97.37^**^
Flower diameter (cm)	14.4^a^ ± 0.5	13.3^c^ ± 1.2	14.0^b^ ± 1.7	-6.34^**^	-8.27^**^	13.7^bc^ ± 1.1	13.8^bc^ ± 1.7	14.3^a^ ± 0.7	-1.43 ^ns^	-3.49^*^
Flower color	white	white	white	—	—	white	white	white	—	—
Stem color	green	green	green	—	—	green	green	green	—	—

**Significant difference with p < 0.01, *Significant difference with p < 0.05, nsNon–significant difference with p < 0.05. Different letters within particular traits in each cross are statistically significant and same letters are statistically non–significant.

The quantification of mid–parent heterosis (MPH) and high–parent heterosis (HPH) in relation to tested traits were varied significantly (*p* < 0.05) between hybrids and their respective parents (Table 1). For plant height, MPH (31.01%) was higher than the HPH (26.61%) in L4–7 hybrid, whereas in hybrid L4–104, MPH (47.79%) was higher than the high–parent heterosis (HPH) (31.34%) (Table 1). For leaf length, MPH (4.32%) was higher than the HPH (–6.45%) in L4–7 hybrid, whereas MPH (46.83%) was higher than the HPH (42.31%) in hybrid L4–104. For days to flowering, MPH (–3.41%) was higher than the HPH (–4.66%) in L4–7 hybrid whereas MPH (–4.44%) was higher than the HPH (–5.67%) in hybrid L4–104. For number of flowers, MPH (94.34%) was higher than the HPH (80.70%) in L4–7 hybrid, whereas MPH (100%) was higher than the HPH (97.37%) in hybrid L4–104. For flower diameter, MPH (–6.34%) was higher than the HPH (–8.27%) in L4–7 hybrid whereas MPH (–1.43%) was higher than the HPH (–3.49%) in hybrid L4–104 (Table 1).

### Illumina paired–end sequencing

**Figure 2:**
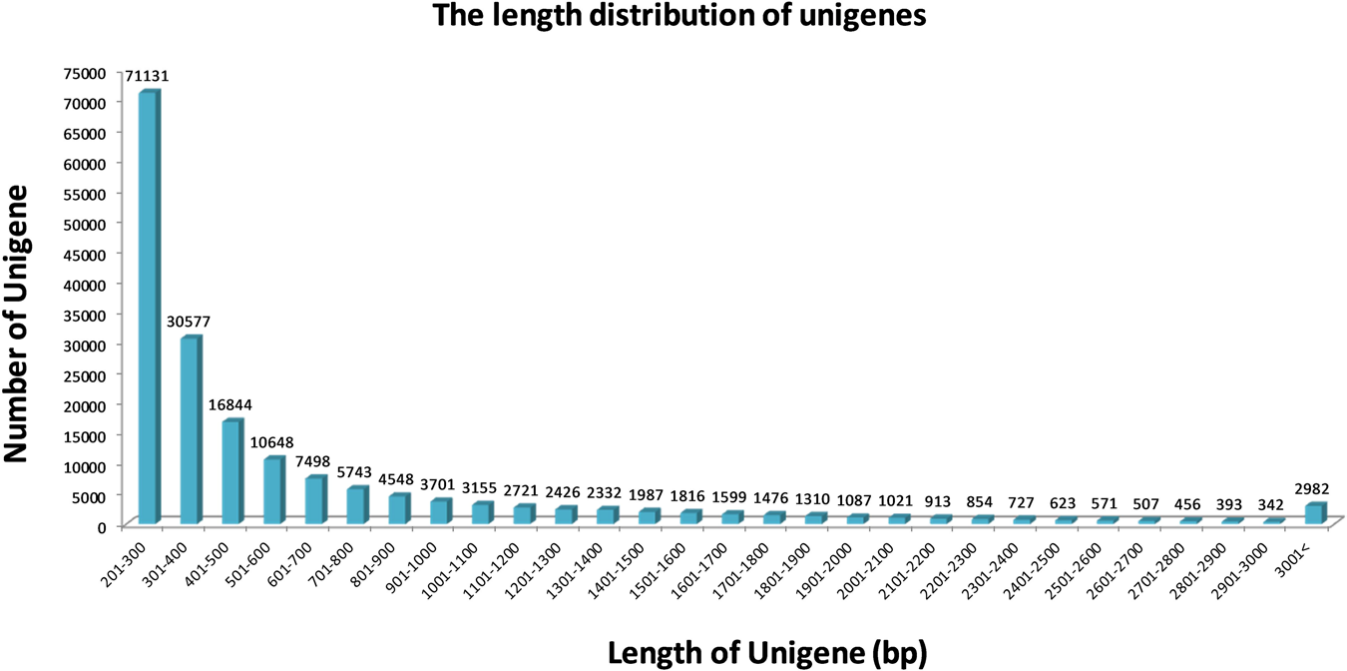
The length distribution of unigenes identified in the transcriptomes of lily plants.

We used RNA–Seq to discover differentially expressed heterotic genes and to investigate the function of those DEGs in hybrids in compared to parents. To identify heterotic transcripts of interest, six cDNA libraries were constructed from leaf RNA samples of six genotypes including two hybrids (L4–7 and L4–104) and four inbred parent lines (L2–4, L2–28, L2–22, and L2–20) of *L*. *longiflorum* and subjected to RNA–Seq analysis on the Illumina HiSeq. 2000 platform. A total of 743,964,980 short reads, each of approximately 100 bp, were generated from the six genotypes (Supplementary Table S2). After stringent quality assessment and data filtering such as removing adaptor sequences and discarding low quality reads, overall 711,948,744 high–quality 100–bp clean reads with a base quality score of 20% were selected from the six genotypes for further analysis. The GC contents of the six libraries were more than 51% (Supplementary Table S2). The parents and their hybrids showed strong correlation with respect to gene expression levels according to Pearsons’s correlation with –1 ≤ r ≤ 1 (Supplementary Fig. S1). The transcripts were then clustered into unigenes. A total of 179,988 unigenes were assembled with a total length of 113,117,791 bp, and the lengths of the transcripts were ranged from 201 to 16,536 with an average of 628 bp (Fig. 2 and Supplementary Table S3).

About 83.72% of the transcripts were in the range of 201–1000 bp (~1 kb), 14.62% transcripts were 1001–3000 bp (1.1 to 3.0 kb) and 1.66% was longer than 3001 bp (>3 kb) (Supplementary Table S3). From the blast results of all 179,988 unigenes in TAIR genome database, finally, 53,209 annotated unigenes were detected with at least one sequence read (Data not shown).

### Identification and analysis of DEGs by RNA–Seq

**Figure 3:**
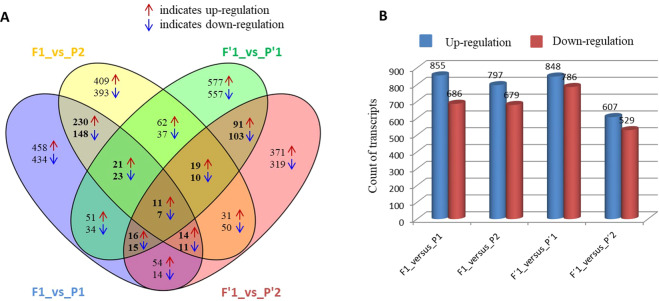
Identification of differentially expressed genes (DEGs) between hybrids and parents. Total numbers of DEG in L4–7_vs_L2–4, L4–7_vs_L2–28, L4–104_vs_L2–22, and L4–104_vs_L2–20 by venn diagram software version 2.1 (3A). Statistics of up– or down–regulated genes between hybrids and parents was shown by colored arrow heads (3A) and next to colored colomns (3B). Bold numerals indicate commonly expressed up– and/or down–regulated DEGs between hybrid (s) and parents (3A).

Putative heterotic DEGs in hybrids over their respective parents were identified using the following criteria, fold change (FC) ≥ 2 for up–regulated and ≤ –2 for down–regulated DEGs. Based on these criteria, a total of 4,327 up– and/or down–regulated transcripts as reliable DEGs were identified from six genotypes involving two crosses of L2–4 × L2–28 (F1, L4–7) and L2–22 × L2–20 (F′1, L4–104) in *L. longiflorum* (Data not shown). The numbers of up– and/or down–regulated DEGs in the hybrid with each parent combination varied considerably (Fig. 3A,B).

Some DEGs were over–expressed either in hybrids or parents. Among 4,327 DEGs, a total of 1,541 DEGs varied from hybrid L4–7 to parent L2–4 (855 up– and 686 down–regulated), 1,476 DEGs varied from hybrid L4–7 to parent L2–28 (797 up– and 679 down–regulated), 1,634 DEGs varied from hybrid L4–104 to parent L2–22 (848 up– and 786 down–regulated), and 1,136 DEGs varied from hybrid L4–104 to parent L2–20 (607 up– and 529 down–regulated) (Fig. 3B). Some DEGs had genotype specific up– and/or down–regulated expression and those were recognized as genotype specific unigenes (Fig. 3A). Venn diagram analysis revealed that overall 737 transcripts showed up and/or down–regulation (highlighted as black bold font) between hybrids and parents in both sets of cross combinations (Fig. 3A). Out of 737 transcripts, a total of 276 up– and 189 down–regulated DEGs (sum of bold font numerals) were commonly observed between L4–7_vs_L2–4 and L4–7_vs_L2–28 whereas 137 up– and 135 down–regulated DEGs (sum of bold font numerals) were commonly observed between L4–104_vs_L2–22 and L4–104_vs_L2–20 (Fig. 3A and Supplementary Table S4A). In hybrids from both cross combinations, 11 up– and 7 down–regulated DEGs were found commonly expressed. Again, 14 DEGs were up– regulated in L4–7 and down–regulated in L4–107 hybrids whereas 2 DEGs were up–regulated in L4–107 and down–regulated in L4–7 hybrids (Fig. 3A and Supplementary Table S4A). Thus, a total of 703 up– regulated and down–regulated DEGs were selected for subsequent analysis (Supplementary Table S4A–C).

To explore and categorize the expression alterations, we classify the commonly expressed 703 DEGs into 12 possible expression patterns for hybrids and parents (Table 2). We observed the notable mode of gene action differences especially in additive gene expression effect (classes 1 and 12) on commonly expressed 703 DEGs between two crosses of hybrids L4–104 and L4–7 (Table 2). In both hybrids, total additive gene effect was 19.35% (Table 2).

**Table 2:** Classification of expression patterns of commonly expressed 703 DEGs in hybrids and their respective parents in *L. formolongi*. DEGs were classified according to the expression levels exhibited by parental and hybrid lines. Additive expression of genes: classes 1 and 12 (blue); dominance expression genes: classes 2, 11, 4 and 9 (green); overdominance expression genes: classes 5, 6, 8, 3, 7 and 10 (red). Classes 5, 6 and 8 represents transgressive upregulation and classes 3, 7 and 10 represent transgressive downregulation ^31^. Diagrams of each class represents the relative expression levels observed in the maternal parent (left point), F1 (middle point), and paternal parent (right point).

Sl. No.	Categories	Expression patterns of DEGs													Total DEGs
		Additivity			Non-additivity										
					Dominance				Overdominance						
					ELD_♂		ELD_♀		Transgressive up–regulation			Transgressive down–regulation			
	Classes	1		12	2	11	4	9	5	6	8	3	7	10	
	Relative expression	♀–F1–♂		♀–F1–♂	♀–F1–♂	♀–F1–♂	♀–F1–♂	♀–F1–♂	♀–F1–♂	♀–F1–♂	♀–F1–♂	♀–F1–♂	♀–F1–♂	♀–F1–♂	
1	Hybrid L4–7 = n, (L2–4 × L2–28)	32		51	2	7	3	7	148	156	56	108	9	124	703
	Sum	83			9		10		360			241			
2	Hybrid L4–104 = n, (L2–22 × L2–20)	121	68		14	13	3	18	132	62	32	128	10	102	
	Sum	189			27		21		226			240			
Average in both (%)		136			18		15.50		293			240.50			
Per cent (%) in both		19.35			2.56		2.20		41.68			34.21			100
					4.76				75.89						
					80.65										

DEGs, differential expressed genes, ♀, maternal parent, ♂, paternal parent, F1, hybrid, ELD, expression–level dominance, Sl, serial, n, the total number of differentially expressed genes in each class, additivity: F1 ≈ 1/2 (♀ + ♂), non-additivity: F1 > 1/2 (♀ + ♂) or F1 < 1/2 (♀ + ♂), ELD_♀: F1 ≈ ♀> ♂ or F1 ≈ ♀ <♂, ELD_♂: F1 ≈ ♂> ♀ or F1 ≈ ♂ <♀, transgressive up–regulation: F1 > ♀ and F1 > ♂, transgressive down–regulation: F1 < ♀ and F1 < ♂.

Non–additivity (80.65%) was mainly classified into dominance and overdominance gene expression effect (Table 2). The dominance effect in both crosses (4.76%) is again subdivided as expression level dominance in paternal effect (ELD_♂) (classes 2 and 11) comprised of 2.56% variation and in maternal effect (ELD_♀) (classes 4 and 9) comprised of 2.20% variation (Table 2). Conversely, noteworthy variation in overdominance effect (75.89%) was observed in L4–7 and L4–104 (Table 2). Hybrids L4–7 and L4–104 showed higher transgressive up–regulation (classes 5, 6, 8) comprised of 41.7% while they showed transgressive down–regulation (classes 3, 7, 10) comprised of 34.2% (Table 2).

### Transcriptome profiles of hybrids and parents

**Figure 4:**
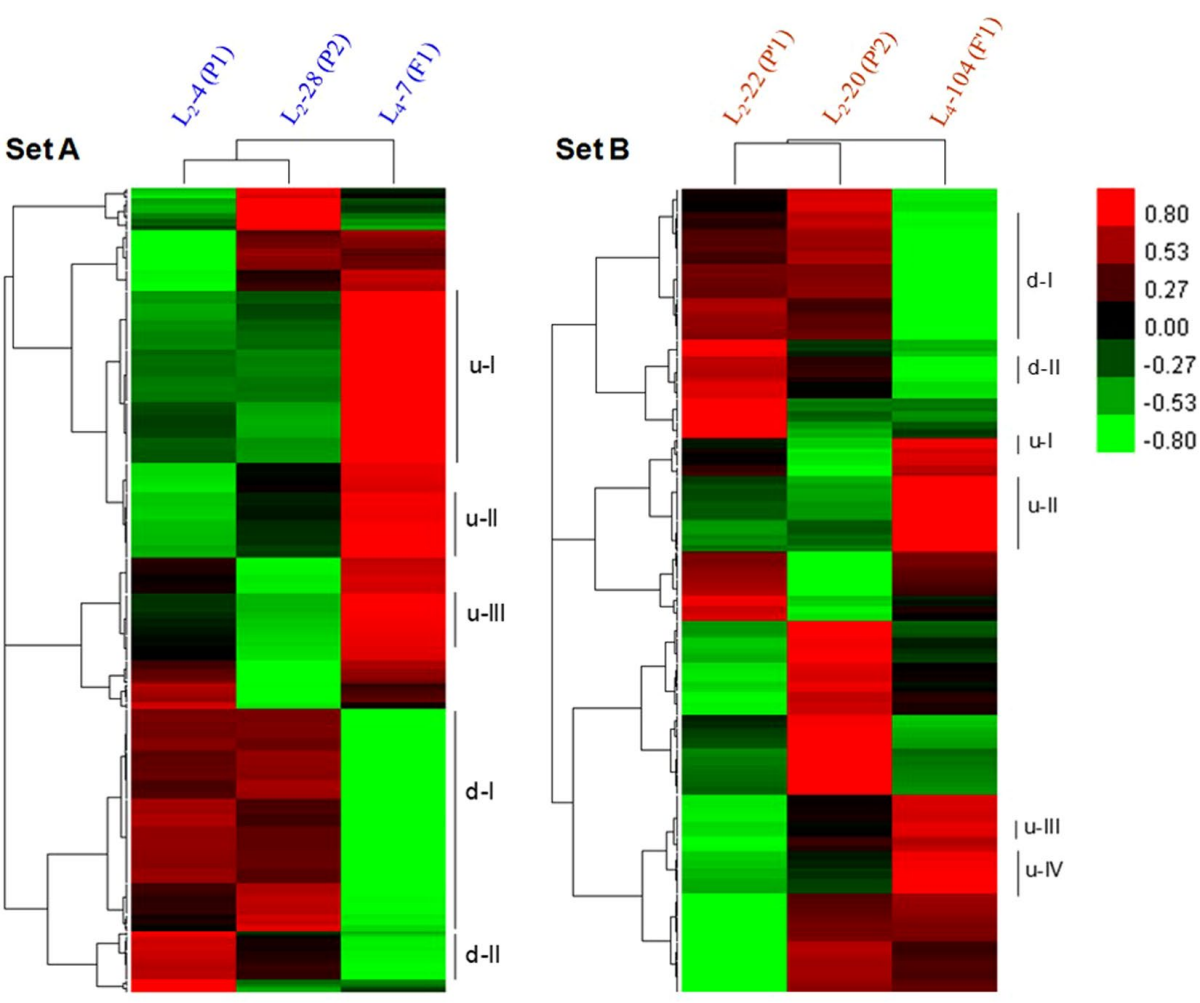
Heat map analysis of functionally characterized DEGs (384) for the four parental inbred lines viz., L2–4 (P1), L2–28 (P2), L2–22 (P′1), and L2–20 (P′2) and two hybrids viz., L4–7 (F1), and L4–104 (F′1). L2–4 (P1), L2–28 (P2) and F1 (L2–4 (P1) × L2–28 (P2) (**A**); L2–22 (P′1), L2–20 (P′2) and F′1 (L2–22 (P′1) × L2–20 (P′2) (**B**). The red color denotes the highly expressed up–regulated DEGs, and the green color denotes down–regulated DEGs with lower expression levels. The gradation from red to green represents the transition from large to small values of a FPKM normalized log_2_ transformed counts. The up– (uI, uII, uIII, uIV) and down– (dI, dII) regulated DEGs are enlisted in Table S5B.

Blast2GO search showed that out of 703 DEGs 384 were functionally characterized, and rests 319 were without any blast hits (Supplementary Fig. S3). Among the 384 DEGs, those were functionally characterized, 260 were functionally annotated, 66 had hits with GO mapped, and 58 had only blast hits (Supplementary Fig. S3). Correlations of 384 functionally characterized up– and down–regulated DEGs between hybrids and their respective parents in two cross combinations were investigated using heat map analysis (Fig. 4 and Supplementary Table S5A).

A total of 139 and 127 DEGs were up– and down–regulated, respectively, in L4–7 hybrid compared to the parental lines L2–4 and L2–28 (Fig. 4A and Supplementary Table S5B). In contrast, 68 and 73 DEGs were up– and down–regulated, respectively, in L4–104 hybrid compared to the parental lines L2–22 and L2–20 (Fig. 4B and Supplementary Table S5B). Based on contrasting expression pattern between hybrids and their respective parents, 12 up– and 16 down–regulated DEGs were identified in both crosses and considered them as universal DEGs in *L*. *longiflorum* (Table 3 and Supplementary Table S5B). Importantly, the up– and down–regulated universal DEGs showed similarity with the different types of proteins and enzymes (Table 3).

**Table 3:** Expression level of functionally characterized universal DEGs in *L. longiflorum*. Hybrid 1, L4–7 (F1); Hybrid 2, L4–104 (F'1).

Genetic hypotheses	DEGs		L4–7_vs_ L2–4	L4–7_vs_ L2–28	L4–104_vs_ L2–22	L4–104_vs_ L2–20	Functional characterization
			Expression level by fold change (FC) value				
Overdominance (1 + 2)	**Up–regulated**	c47423g1i1	**2.75**	**2.69**	**2.21**	**2.14**	ABC transporter A family member 7–like
Dominance (1) Overdominance (2)		c54944g1i1	**2.15**	1.60	**2.69**	**2.22**	ABC transporter C family member 10–like
Additive (1) Overdominance (2)		c60389g1i1	1.23	1.35	**2.75**	**3.69**	B3 domain–containing Os04g0386900–like
Additive (1) Overdominance (2)		c60389g1i2	1.18	1.14	**2.35**	**3.02**	B3 domain–containing Os04g0386900–like
Overdominance (1 + 2)		c49702g1i1	**2.96**	**2.77**	**4.85**	**3.18**	monothiol glutaredoxin–S11
Dominance (1) Overdominance (2)		c52443g1i1	**2.27**	1.75	**7.99**	**7.26**	trans–resveratrol di–O–methyltransferase–like
Overdominance (1) Additive (2)		c57602g1i1	**2.76**	**3.22**	1.16	1.24	auxin efflux carrier component 8
Overdominance (1 + 2)		c59275g1i2	**2.20**	**2.16**	**5.80**	**4.39**	probable mannitol dehydrogenase
Overdominance (1) Additive (2)		c62533g1i1	**2.46**	**2.13**	1.25	1.27	tRNA–dihydrouridine(16 /17) synthase [NAD(P)( + )]–like
Additive (1) Overdominance (2)		c63435g1i2	1.29	1.19	**3.97**	**3.22**	very–long–chain enoyl– reductase–like
Additive (1) Overdominance (2)		c64671g4i2	1.63	1.39	**2.25**	**2.38**	disease resistance RPP13/1
Dominance (1) Overdominance (2)		c60887g1i2	1.87	**2.21**	**2.47**	**2.79**	conserved hypothetical protein
Dominance (1) Overdominance (2)	**Down–regulated**	c52384g1i2	-1.97	**-2.96**	**-2.38**	**-2.18**	vicilin–like antimicrobial peptides 2–2
Overdominance (1 + 2)		c58513g1i1	**-2.63**	**-4.58**	**-2.84**	**-3.66**	vicilin–like antimicrobial peptides 2–2
Overdominance (1) Dominance (2)		c47447g1i1	**-4.33**	**-3.18**	-1.92	**-2.04**	auxin–responsive SAUR68–like
Overdominance (1 + 2)		c63663g3i2	**-2.84**	**-2.49**	**-2.06**	**-2.11**	auxin–responsive SAUR68–like
Overdominance (1 + 2)		c47368g1i1	**-3.74**	**-2.04**	**-2.32**	**-2.88**	probable xyloglucan-glycosyltransferase 12
Overdominance (1 + 2)		c59731g1i1	**-2.76**	**-4.20**	**-3.94**	**-2.22**	probable WRKY transcription factor 70
Additive (1) Overdominance (2)		c47856g3i1	-1.78	-1.66	**-2.37**	**-2.39**	regulation of nuclear pre–mRNA domain–containing 1A–like
Overdominance (1 + 2)		c37944g1i1	**-2.41**	**-5.84**	**-3.36**	**-2.88**	heavy metal–associated domain containing
Additive (1) Overdominance (2)		c55548g2i1	-1.38	-1.45	**-2.47**	**-2.08**	oxidative stress isoform 1
Additive (1) Overdominance (2)		c56718g1i1	-1.74	-1.59	**-2.60**	**-2.71**	carboxypeptidase 2
Additive (1) Overdominance (2)		c57368g1i1	-1.34	-1.19	**-2.26**	**-2.03**	pentatricopeptide repeat–containing At1g62350–like
Overdominance (1) Additive (2)		c60709g3i1	**-3.59**	**-4.70**	-1.19	-1.13	chloroplastic–like
Overdominance (1) Additive (2)		c80430g1i1	**-2.91**	**-4.30**	-1.27	-1.27	lysine histidine transporter–like 8
Overdominance (1) Dominance (2)		c96948g1i1	**-2.95**	**-6.14**	**-2.62**	-1.99	naringenin,2–oxoglutarate 3–dioxygenase–like
Overdominance (1 + 2)		c13258g1i1	**-2.33**	**-2.74**	**-3.25**	**-2.23**	hypothetical protein PHAVU_005G042200g
Overdominance (1) Additive (2)		c22243g1i1	**-2.83**	**-2.58**	-1.59	-1.18	hypothetical protein MIMGU_mgv1a014176mg

Bold numerals indicate significant expression level of up– (+) and down– (–) regulated differentially expressed genes (DEG).

### Functional classification by gene ontology

**Figure 5:**
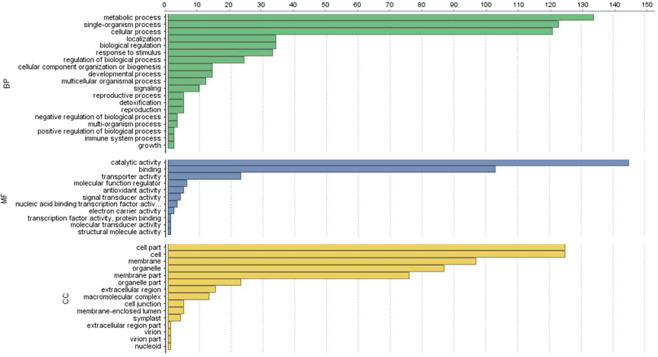
Comparative Gene Ontology (GO) classifications of commonly expressed functionally annotated DEGs from two cross combinations in the lily transcriptome. The genes corresponded to three main categories, cellular component (CC), molecular function (MF), and biological process (BP).

Gene Ontology (GO) categories revealed that functionally annotated 260 DEGs (36.98%) with coding regions bear at least one functional group (Supplementary Table S6). The 260 proteins were assigned into 45 GO functional groups within three main categories, molecular function (sub–categories, 11), biological process (sub–categories, 19), and cellular components (sub–categories, 15) (Fig. 5). In the cellular component category, cell and cell parts followed by membrane, organelle and membrane part were the predominant subcategories. In the molecular function category, catalytic activity and binding followed by transporter activity were the predominant subcategories. In the biological process category, metabolic process, single organism process, and the cellular process followed by localization, biological regulation, and response to stimulus were the predominant subcategories (Fig. 5).

### Kyoto encyclopedia of genes and genomes (KEGG) pathway mapping

KEGG pathway–based analysis was performed using Blast2GO software to interrogate the KEGG database for further deepen our understanding of the biological functions and interactions of genes. All 260 functionally annotated transcripts sequences were selected and assigned to the reference canonical pathways in KEGG. A total of 45 DEGs were distributed to 59 different metabolic pathways in the KEGG database (Table S7). We observed that each of 15 KEGG pathways comprised more than one DEG while each of the remaining 44 pathways contained only one DEG (Supplementary Table S7). The most enriched KEGG Orthology terms (ko–term) with the highest levels of gene representation were biosynthesis of antibiotics (ko01055, 8 transcripts) followed by phenylpropanoid biosynthesis (ko00940, 6 transcripts), carbon fixation in photosynthetic organisms (ko00710, 5 transcripts), methane metabolism (ko00680, 5 transcripts), pyrimidine metabolism (ko00240, 5 transcripts), purine metabolism (ko00230, 5 transcripts), fructose and mannose metabolism (ko00051, 4 transcripts), pentose phosphate pathway (ko00030, 4 transcripts glycolysis/gluconeogenesis (ko00010, 4 transcripts), thiamine metabolism (ko00730, 3 transcripts), sulfur metabolism (ko00920, 3 transcripts), starch and sucrose metabolism (ko00500, 2 transcripts), drug metabolism cytochrome P450 (ko00982, 2 transcripts), pentose and glucuronate interconversions (ko00040, 2 transcripts), and other glycan degradation (ko00511, 2 transcripts) (Supplementary Table S7).

### Validation of DEGs by quantitative real–time PCR

**Figure 6:**
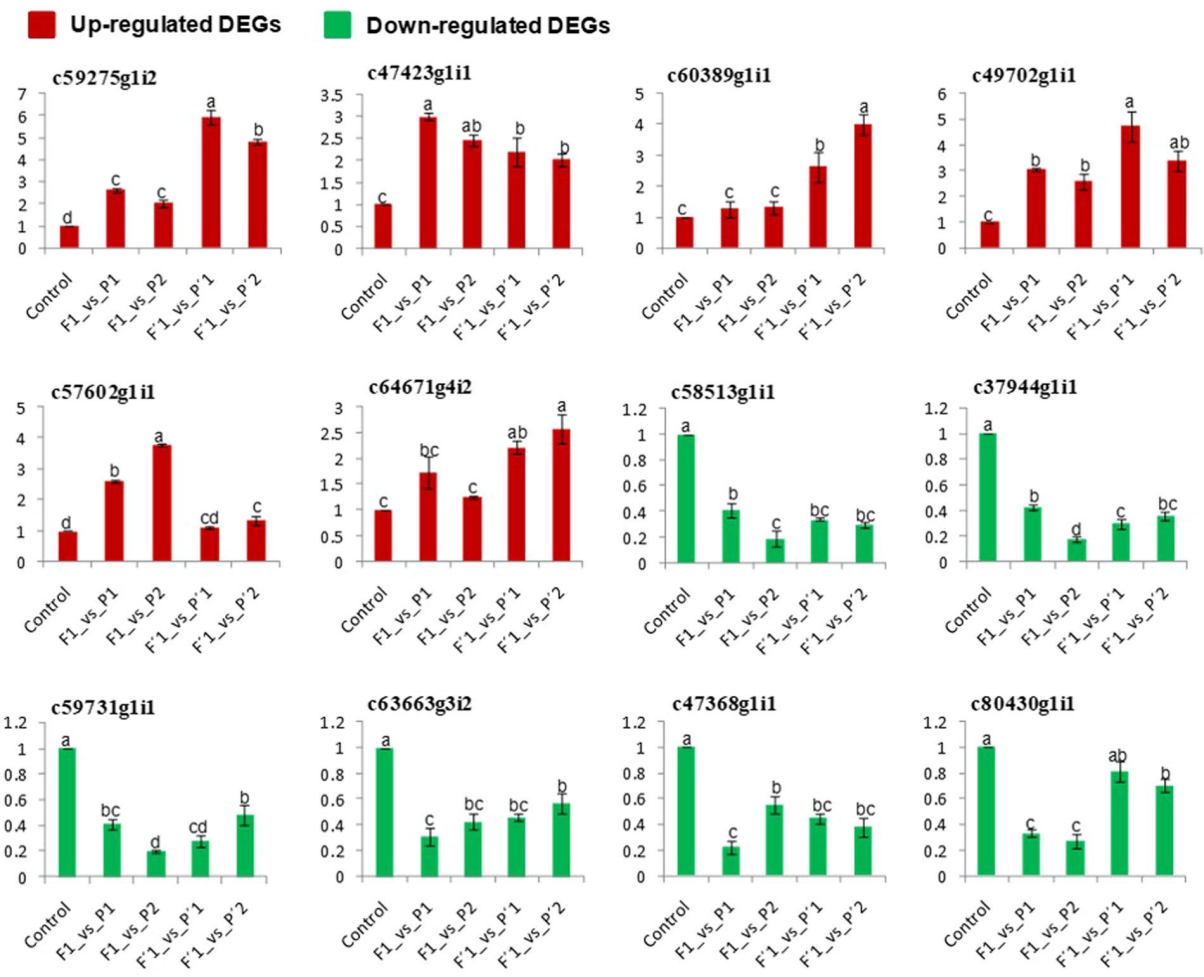
Validation of differential expression of 12 universal genes using real–time quantitative PCR (qPCR). The relative transcript levels were normalized with *Lf–actin* as the standard. Red and green colored bars denote up– and down–regulated DEGs, respectively. The error bars indicate the standard error of the mean of three independent replicates. Different lower case letter (a, b, c, d) indicates the significant difference in F1_vs_P1 (L4–7_vs_ L2–4), F1_vs_P2 (L4–7_vs_ L2–28), F′1_vs_P′1 (L4–104_vs_ L2–22), and F′1_vs_P′2 (L4–104_vs_ L2–20) compared to control at *p* < 0.05.

A subset of the 12 DEGs (6 up– and 6 down–regulation types, yellow highlighted in Table 3) were selected for the verification of the expression patterns using real–time quantitative PCR (qPCR) validation (Supplementary Table S1 and Fig. 6). We compared the transcript profiles obtained from qPCR with those generated from RNA–Seq analysis from the six genotypes of *L. longiflorum*. The data in Fig. 6 confirmed that almost all 12 DEGs displayed the expression patterns consistent well with expression levels obtained from RNA–Seq analysis.

## Discussion

Heterosis is a widely exploited phenomenon in plant breeding in cross–pollinated crops. In general, the higher the heterosis and heritability, the simpler the selection process and greater the response to selection. In this study, the recorded higher plant height, leaf length, and number of flowers but lower days to flowering and flower diameter in both hybrids in contrast to their parents could be attributed by the genetic make–up of the genotypes. Transcriptome analysis identified some universally expressed DEGs in both hybrids. The functional proteins associated with those universally expressed DEGs might be largely regulating heterosis in both hybrid combinations.

This study investigated seven phenotypic traits including plant height, leaf length, flowering time, number of flowers, and flower diameter that showed hybrid vigor, either mid-parent heterosis or high-parent heterosis, However, only leaf sample was taken from the elongating leaves before any flower appearance for RNA-seq, and we did not look at the transcriptome from other organs like flower. As elongation of leaf was associated with plant height we therefore believe that the DEGs expressed in leaves could be associated with increasing plant height in hybrids^51^. Nevertheless, collection of leaf samples at different growth stages, e.g., two or three growth phases, certainly generate additional sets of RNAseq data. Thus, it makes possible to compare the variable DEGs from the different growth stages. The results obtained from such a comprehensive experiment would provide a confirmatory picture to discerning the genes responsible for heterosis. However, in this study, samples were collected from the 2^nd^ and 3^rd^ youngest leaves those were still elongating. There were more than 20 live leaves per plant at the sampling time and the samples were taken from those plants that did not produce any flower yet (Fig. 1). Leaf elongation and increasing plant height in lily is a dynamic process. Leaf that initiate at the axis of the whorl dynamically undergoes cell division, elongation, expansion, maturation and senescence. Every leaf at any developmental stage of a lily plant pass through those processes. We therefore believe that an elongating leaf at any particular vegetative growth phase of lily plant may produce similar transcriptomic profiling with some variations related to plant development. In our study, both sampled-leaves and sampling plants were still elongating. We therefore believe that the differentially expressed genes between parents and hybrids should be responsible for controlling the plant height and leaf elongation. Thus the transcriptomic profiles obtained in this study are expected to represent plant height. However, the expression of genes related to flowering time, number of flowers, and flower diameter is a subject of further investigation.

Transcriptome analysis as a powerful tool facilitates the identification of DEGs with their expression level and regulatory mechanisms. Previous investigations identified various heterotic DEGs related to a variety of traits between hybrids and parents in plants and animals^13,31,52,53^. Heterotic genes are robust due to multiple allelic combinations between genomes of two parents in both cross and reciprocal cross combinations suggesting that major genes genetically govern this phenomenon^31,54^. Divergent patterns of DEGs between hybrid and its inbred parents play a significant role in hybrid vigor or heterosis^13,18,20,31,55–59^. RNA–Seq generated approximately 711 million high–quality 100–bp paired–end reads from the leaves of six genotypes, which were almost double compared to rice root transcriptome^13^. In addition, 53,209 annotated transcripts were identified that was 1.37 times higher compared to rice root transcriptome (38,872 annotated transcripts)^13^. A total of 4,327 DEGs were selected based on fold change value in hybrid compared to inbred parents indicating that a large number of genes could be functionally associated with hybrid vigor in lily. Out of 4,327, 703 commonly up– or down–regulated DEGs were identified between hybrids and parents in both crosses (Fig. 3). Among them, 384 functionally annotated DEGs were identified using Blast2GO analysis (Supplementary Table S5A).

One of the main interest of this study is to compare the levels of additive and non-additive gene expression in two hybrids with varying levels of heterosis for leaf length which is likely associated with plant height^51^. Interestingly, less than 20% DEGs showed additivity on an average indicating that non-additive gene interactions have major control in heterosis (Table 2)^21,36^. Moreover, between dominance and overdominance non-additive interactions, the overdominance effect was vital as it controls more than 75% gene effect (Table 2). Both transgressive upregulation of DEGs and down-regulation of DEGs have major effect on heterosis (Table 2).

Importantly, heat map analysis revealed that 12 up– and 16 down–regulated universal DEGs in both sets of hybrids were remarkably down– and up–regulated, respectively, in parental lines indicating those universal genes might have a more certain functional role in plant height in comparison with remaining DEGs pool (Fig. 4). Moreover, among the 28 universally up- and down-regulated genes, nine genes exhibited overdominance effect in both hybrid combinations and remaining 19 genes showed overdominance effect either in one of two hybrid combinations (Table 3). This predicted genetic analysis indicated that majority of the 28 universal DEGs have overdominance effect to bring about heterosis for plant height in Easter lily, in this study. In *Nicotiana tabacum*, transcriptome analysis revealed that the heterosis for nicotine biosynthesis is critically controlled by overdominance effect of up-regulated genes related to nicotine anabolism and transport^29^. Functional annotation of these up– and down–regulated DEGs revealed that they were very close to the various classes of proteins and enzymes (Table 3). These common protein classes include ABC transporter family member–like protein and B3 domain–containing proteins among others.

Among 12 up–regulated transcripts, two transcripts showed similarity in functional annotation with the ABC transporter family member–like protein (Table 3) that is actively involved in DNA repair, RNA translocation, and active transport of a wide variety of substrates in cells as well as leading to coping with biotic and abiotic stresses in adverse conditions^60^. Both of these two ABC transporter family members exhibited overdominance in F'1 (L4–104) hybrid but one gene showed dominance and another gene showed overdominance in another hybrid, F1 (L4–7), indicating that non-additive gene interaction in involved in this case (Table 3). Another two transcripts showed similarity with the abscisic acids and auxins B3 domain–containing proteins that take part in plant growth and seed maturation process^61,62^. A single transcript showed similarity with the monothiolglutaredoxin–S11 (Table 3) which has anti–oxidative protective roles in the cellular response against reactive oxygen species in plants^63^. Another single transcript showed similarity with the synthase–like and trans–resveratrol di–O–methyltransferase–like enzyme that protect plants against pathogens by catalyzing the biosynthesis of plant phytoalexins^64,65^. Both of these two B3 domain-containing proteins exhibited additive effect in F1 (L4–7) hybrid but overdominance effect in F'1 (L4–104) hybrid (Table 3). Other individual transcripts showed similarity with the different families such as auxin efflux carrier family component 8, probable mannitol dehydrogenase, tRNA–dihydrouridine synthase, very–long–chain enoyl–ACP (acyl carrier protein) reductase enzymes (EC 1.3.1.9), disease resistance *RPP13/1* gene (Table 3). These genes play an important role in auxin–related plant growth and development^66–68^, pathogen–induced stress resistance^69–72^ and fatty acid biosynthesis^73^. Thus, the twelve commonly expressed DEGs and their associated proteins likely function to tackle plant height under various biotic and abiotic stress environments and excessive transcript abundance of those genes in hybrids eventfully might help in maintaining hybrid vigor largely through overdominance gene effect.

Among 16 down–regulated transcripts, two transcripts showed similarity with the auxin–responsive SAUR68–like (SMALL AUXIN UP RNAs) genes (Table 3) that enhance auxin–induced growth in hypocotyls, inflorescence, stems, petals and stamen filaments^74^. One transcript showed similarity with the heavy metal associated domains containing protein that involved in heavy metal transport and heavy metal homeostasis in plants^75,76^. Another single transcript showed similarity with the probable xyloglucan-glycosyltransferase 12 enzyme (Table 3) that functions to swell the cell wall for plant growth process^77^. Thus any DEGs associated with biosynthesis of xyloglucan-glycosyltransferase 12 enzyme might be associated with cell division of plants that is closely related to increasing both leaf length and plant height and the gene effect could be heterotic in hybrids largely due to overdominance (Table 3). Another transcript showed similarity with the regulation of nuclear pre–mRNA domain–containing 1A–like (RPRD1A–like) protein that is involved in a cell–cycle and dimerization process^78^. A single transcript showed similarity with the vicilin–like antimicrobial peptides 2–2 which functions in defense related responses against a wide range of pathogens^79^. Another transcript showed similarity with the oxidative stress isoform 1 (Table 3) which is involved in generating ROS particularly H_2_O_2_ for plant defense mechanism^80,81^. Other individual transcripts were from different families such as pentatricopeptide repeat (PPR), WRKY transcription factor 70, chloroplastic–like gene, lysine histidine transporter–like 8, naringenin, 2–oxoglutarate 3–dioxygenase–like enzyme etc (Table 3). These genes have vital role in post–transcriptional processes^82,83^, biotic and abiotic stress tolerance^84^, growth and developmental process^85^ and biosynthesis of proteins and secondary metabolites^86,87^. In 16 universally down-regulated genes, at least one hybrid showed overdominance although four of them exhibited additive effect in F1 (L4–7) and three of them exhibited additive gene in F'1 (L4–104) (Table 3). Thus, similar to up–regulated DEGs, the sixteen commonly expressed DEGs and their associated proteins might also be involved to tackle plant height in hybrids largely through overdominance effect of genes under various biotic and abiotic stress environments, however, excessive transcript shortage of those genes in hybrids eventfully might reduce the hybrid vigor.

Finally, the expression levels of 12 universal up– and down–regulated transcript generated from RNA–Seq analysis were verified by qPCR and the expression patterns of all the DEGs tested were consistent with the RNA–Seq results indicating reliability of the RNA–Seq data from the six genotypes of *L. longiflorum* (Fig. 6). Taken together, the genes identified herein might be useful for creating genetic diversity with increased hybrid vigor in *L*. *longiflorum*. However, further experiments using genes with specific trait of interest are needed to confirm the heterotic association between the gene expression patterns and the target agronomic traits in lily.

In this study, the majority of the commonly expressed DEGs revealed non–additive effect especially overdominance (transgressive up– and down–regulation) mode of gene expression patterns in both cross combinations (Table 2). Thus, overdominance mode of gene expression patterns might contribute more than the additive as well as dominance effects to intraspecific heterosis between hybrids and parents in *L. longiflorum*^52^.

Functional analysis placed the 260 DEGs into 45 subcategories (Fig. 5). GO terms representing biological processes, accounted for almost half of the 45 categories, indicating a diverse range of biological processes especially metabolic process, single–organism process, and cellular process might play major role in increasing plant height of lily hybrids (Fig. 5). Previous reports showed that biological processes including metabolic process and cellular process are related to root development and yield of rice as extensive metabolic activities were taking place in roots of hybrid plants in both vegetative and reproductive stages^13^. KEGG analysis revealed that 45 functional DEGs contributed to 59 different metabolic pathways possibly regulated hybrid vigor in lily (Fig. 5 and Supplementary Table S7). Reports showed that differentially expressed heterotic genes in rice control hybrid vigor by controlling metabolic pathways especially carbohydrate metabolism in root tissues for root elongation^13^. However, some DEGs were associated with more than one metabolic pathway indicating their overlapping functions in different metabolic activities and might be candidate heterotic genes for the growth, development, and stress–related mechanism in *L*. *longiflorum* (Supplementary Table S7).

The results presented in this study and the discussion has been made mostly focused a common set of DEGs in the two hybrids which could be of interest in regulating heterosis of leaf elongation and plant height. The confirmation of the specificity of genes responsible for heterosis of leaf elongation and plant height is subject of further investigation. As majority of DEGs showed non-additivity and specifically overdominace effect, we therefore comment on that overdominace gene interaction might be responsible of heterosis related to leaf elongation and plant height in Easter lily.

## Conclusions

In summary, we observed higher MPH for positive heterotic traits such as plant height, leaf length, and number of flowers than HPH whereas HPH was higher for the negative heterotic trait flowering time in *L. longilforum*. Blast2GO analysis of 703 DEGs revealed that 384 DEGs were functionally characterized. The expressed DEGs might be associated with overdominance effects in hybrids to show heteroses in lily. Heat map analysis of 384 DEGs revealed that 12 and 16 DEGs were up- and down-regulated, respectively, in both sets of hybrids and showed similarity with different types of proteins and enzymes. The major protein families possibly involved in heterosis are ABC transporter A family member–like, B3 domain–containing, disease resistance RPP13/1,auxin–responsive SAUR68–like, and vicilin-like antimicrobial peptides 2–2 proteins etc. In addition, GO analysis indicated that biological process might contribute more in heterotic related genes expression compared to other processes. Furthermore, KEGG pathway–based analysis revealed that some DEGs were involved in more than one metabolic pathways indicating that some heterotic related genes might be functionally redundant in *L. longiflorum*. Validation of up– and down regulated universal DEGs showed that the expression trends were consistent with the RNA–Seq data suggested the reliability of the transcriptome data in *L. longiflorum*. This study identified some important protein families associated with universally up– or down–regulated DEGs. The results of this study advance the molecular underpinning of heterosis for plant height in lily plants.

## Materials and Methods

### Plant materials and growing conditions

Seeds of two hybrids (L4–7, L2–4 × L2–28 and L4–104, L2–22 × L2–20) with their four inbred parents (L2–4, L2–28, L2–22 and L2–20) of Lilium longiflorum (Easter lily) were obtained from the Department of Floriculture, Korea National Agricultural College, Hwaseong, Korea. Seeds of hybrids and inbred lines were sown in the plastic pots (BN–150, 13.5 cm × 10.5–15 cm) containing sterilized soil mixture for germination. Plants were grown under greenhouse environmental condition with 18 ± 2 °C temperature and 80–85% relative humidity. Leaves (2^nd^ and 3^rd^) of four months–aged plants, that did not produce any flower yet, were sampled with three replicates. Collected samples were immediately transferred to liquid nitrogen and kept at −80 °C for subsequent analysis.

### RNA isolation, cDNA library construction, and RNA–sequencing

RNeasy mini kit (Qiagen, Hilden, Germany) was used for total RNA isolation from leaf samples (2^nd^ and 3^rd^) of three replicates from each of six genotypes following the manufacturer’s guidelines. The quality of isolated RNA was checked using NanoDrop ND–1000 spectrophotometer (NanoDrop Technologies, Wilmington, DE, USA). DNA was removed from the samples using RNase–free DNase (Promega, Madison, WI, USA), also according to the manufacturer’s instructions. The integrity of RNA was checked by agarose gel electrophoresis^88^. cDNA synthesis was performed using equal quantities of high–quality RNA from each sample following IlluminaTruSeq^TM^ RNA sample preparation kit. Sequencing libraries were generated according to manufacturer’s guidelines (Illumina, San Diego, CA) for cluster analysis. Two consecutive purification steps were performed using poly–T oligo–attached magnetic beads for the isolation of poly–A–containing mRNA from the total RNA and then they were fragmented using an RNA fragmentation kit. Synthesis of first strand cDNA was done using reverse transcriptase and random primers whereas second strand cDNA was synthesized by DNA polymerase I and RNase H. After second strand cDNA synthesis and adaptor ligation, cDNA fragments of 200–bp were separated by gel electrophoresis. The cDNA fragments were subsequently amplified with PCR primer cocktail with minimum cycles to avoid skewing the representation of the library. The quantity and quality of amplified cDNA library were controlled by Macrogen (Seoul, South Korea). Following loading of amplified products into the Illumina HiSeq. 2000 instrument, they were exposed to automated cycles of paired–end–sequencing (2 × 100 bp). The processing of fluorescent images into sequences, base–calling and quality value calculations were carried out using the Illumina data processing pipeline version 1.8 (Solexa’s Sequencing–by–Synthesis).

### De novo assembly and assessment of differential gene expression

High–quality reads were filtered by eliminating low–quality reads with Q < 20 from the raw reads. To perform quality trimming and removal of adapter, fast and multi–threaded command line tool ‘trimmomatic’ was used (http//www.usadellab.org/cms/?page=trimmomatic)^89^. Further, high–quality reads (transcriptome) were assembled by Illumina/Solexa with *de novo* using trinity program (http//trinityrnaseq.sourceforge.net)^90^ and were quantified of transcript abundances using RSEM (RNA–Seq by Expectation–Maximization) (v1.2.15) (http://deweylab.biostat.wisc.edu/rsem/)^91^. As lily has no reference genome, therefore, RSEM was used for quantification. Transcript expression level was quantified in terms of FPKM (Fragments Per Kilobase of transcript per Million mapped reads) values ranging from > 0 to over 10^4^, and estimated fold change (FC) (Macrogen, Seoul, Korea). Expression of the transcript that satisfied with FC ≥ 2 and FC ≤ –2 was considered as significantly up– and down–regulated DEGs, respectively at least one of total comparisons (Macrogen, Seoul, Korea). The transcriptome sequences of *L. longiflorum* (Easter lily) was deposited in our internal lily database^92^.

### Identification of DEG and cluster analysis

Commonly expressed up– and down–regulated DEGs between hybrids and parents was selected using the Venny diagram (v2.1.0)^93^. Differential up– and down–regulation of annotated transcripts between hybrids and their parents were analyzed using cluster analysis. The cluster of FPKM–normalized expression values for each transcript was made with the cluster software version 3.0 followed by Java TreeView to visualize the results^94^.

### Functional annotation

Blast (blastx) search was conducted to identify functionally annotated DEGs protein against the GenBank non–redundant (NR) database (http://www.ncbi.nlm.nih.gov/), SWISSPROT database (http://www.ebi.ac.uk/swissprot/), Kyoto Encyclopedia of Genes and Genomes (KEGG) database (http://www.genome.jp/kegg/) and Clusters of Orthologous Groups (COG) of protein databases with an E–value of 1e^–5^. Unique transcripts were functionally classified according to gene ontology (GO) using TAIRGO slim of Blast2GO software version 3.3 (https://www.blast2go.com/) and plotted based on their distribution^95^. Association between unique transcripts and Kyoto Encyclopedia of Genes and Genomes (KEGG) pathways was performed using blastp against the KEGG database^96^.

### Phenotypic traits and DEGs expression pattern measurements

Plant height (cm), leaf length (cm), the number of flowers, flowering duration (days), and flower diameter (cm) of the six genotypes were recorded. Calculation of mid–parent heterosis (MPH) and high–parent heterosis (HPH) were performed using the following formulas, MPH = (F1 − MP)/MP in % and HPH = (F1 − HP)/HP in %, where F_1_ denotes the average performance of the hybrid, MP denotes the average performance of the two parents, and HP denotes the average performance of the better–parent between two parents. The mode of gene expression patterns including additivity and non–additivity were performed based on Rapp *et al*.^97^, Zhang *et al*.^52^, and Wu *et al*.^31^. The relative expression level of DEGs were categorized into additive and non-additive (dominant and overdominant) classes based on Wu *et al*.^31^. The relative expression levels of DEGs in between two parents were classified as additive. The relative expression levels of DEGs similar to the dominant parent was classified as dominance. The relative expression levels of DEGs either higher (transgressive upregulation) or lower (transgressive downregulation) than both parents were classified as overdominance.

### Validation of RNA–Seq by qPCR

The quantitative real–time PCR (qPCR) analysis was carried out for the further verification of RNA–Seq data using gene–specific primer sets (Supplementary Table S1). The details of plant culture, sample collection, and RNA extraction have been described in sections 2.1 and 2.2. The cDNA synthesis was carried out for three independent biological replicates against each parent and hybrid genotype. The cDNA was synthesized by Superscript III First–Strand synthesis kit (Invitrogen, California, USA) following the manufacturer’s manual. The qPCR reaction mixture consisted of 1 µL cDNA in a 20 µL reaction volume employing 2× qPCR BIO SyGreen Mix Lo–Rox SYBR Green Super–mix with ROX (PCR Biosystems Ltd., London, UK). There were three technical replicates against each biological replicate for qPCR analysis. The primers used for qPCR were designed by primer premier 3.0 (Premier, Canada) (http://bioinfo.ut.ee/primer3–0.4.0/) and those were synthesized by Macrogen Company (Seoul, South Korea). The thermal cycling conditions for qPCR comprised of pre–incubation at 95 °C for 10 min, followed by 3 step amplifications at 95 °C for 20 s, 58 °C for 20 s, and 72 °C for 25 s for 40 cycles. The melting temperature comprised of 95 °C for 10 s, 65 °C for 60 s, and 97 °C for 1 s as a default setting. For quantification (Cq), the fluorescence was measured at the end of each cycle, and three technical replicates (*N* = *3*) were pooled. Using LightCycler96 (Roche, Germany), amplification, detection, and data analysis were conducted. The *Lf–Actin* from *L. longiflorum* (DQ019459), as an internal control, was used to normalize the gene expression level^98^. The 2^−∆∆ct^ method^99^ was used to calculate the relative gene expression level.

### Statistical analysis

The phenotypic data were analyzed following Zhai *et al*.^13^ and Ding *et al*.^34^. Analysis of variance (ANOVA) was conducted following a generalized linear model using Statistical Analysis System (SAS) software version 9.1 (SAS Inst. Inc., Cary, NC)^100^ to determine the significance of variation for phenotypic traits and qPCR expression level between hybrids and parents.

## Supplementary information

Supplementary information.
