# Supplementary material for: Transcriptome Analysis by RNA–Seq Reveals Genes Related to Plant Height in Two Sets of Parent-hybrid Combinations in Easter lily (Lilium longiflorum)
Source: Sci Rep. 2020 Jun 3;10:9082. doi: 10.1038/s41598-020-65909-x (PMC7270119; doi:10.1038/s41598-020-65909-x)
Supplement: Supplementary file 1 — Supplementary information. [file 41598_2020_65909_MOESM1_ESM.zip › Supplementary files/Table S1.docx]

Table S1. Primer design for quantitative real-time PCR (qPCR) of commonly expressed DEGs from RNA sequencing data in *L*. *longiflorum*

| Transcript id | Forward (5–3´) | Reverse (5–3´) | Annealing Temp. (° C) | Product size  (bp) |
| --- | --- | --- | --- | --- |
| c64671g4i2 | CTTGTTTCCTCGGCTTGATA | AAGTCGGTGAGCAACTCAAG | 58 | 186 |
| c60389g1i1 | CAGCGCTTCACTACTTCCAT | AGGGCAGTTTCTGGACTTCT | 58 | 184 |
| c57602g1i1 | AGTGATGGACCAGCAGTAGC | CTCCCTCCCATTGTTTACCT | 58 | 163 |
| c49702_g1_i1 | ATTTCGTCAGAACCAGCAAG | GTGATAAAGCCCGATGATTG | 58 | 179 |
| c47423_g1_i1 | GGCAATCATCCTCACAACTC | CTTCTCCGCCTCTTCTTCTT | 58 | 181 |
| c59275_g1_i2 | CACATTGAGCCCAAAGTTCT | TCCTTACATACGGTGCCATT | 58 | 185 |
| c59731g1i1 | TTGCAGTCCTCATCCAATTT | TCGCGTTATAGATCCGTTTC | 58 | 199 |
| c37944_g1_i1 | CCAGCTCCAGAGGATTTACA | AGAGCTTGCAGTGGGTAATG | 58 | 174 |
| c58513_g1_i1 | TCATTTTCCTCGAACGAGTC | TCATTGGTGGAGGCATCTAT | 58 | 180 |
| c80430g1i1 | GCAAGGGTAAGCAAAAGTCA | ATGCCGATCTTTGACAACAT | 58 | 195 |
| c63663_g3_i2 | GCATTCATGAGGATGTTGGT | GAATTTGGATTACGGCATGA | 58 | 181 |
| c47368_g1_i1 | ACCCTACTAGAACGGCGACT | GTTGCCAAAATATCGGTTTG | 58 | 172 |

Here, Red and green colors denote up- and down-regulated DEGs, respectively.
