# Supplementary material for: Transcriptome Analysis by RNA–Seq Reveals Genes Related to Plant Height in Two Sets of Parent-hybrid Combinations in Easter lily (Lilium longiflorum)
Source: Sci Rep. 2020 Jun 3;10:9082. doi: 10.1038/s41598-020-65909-x (PMC7270119; doi:10.1038/s41598-020-65909-x)
Supplement: Supplementary file 1 — Supplementary information. [file 41598_2020_65909_MOESM1_ESM.zip › Supplementary files/Table S2.docx]

Table S2. Quality of RNA sequencing data

| **Sample** | **Total bases** | **Read count** | **GC (%)** | **Q20 (%)** |
| --- | --- | --- | --- | --- |
| **Raw data** | | | | |
| L_2_-4 (P1) | 12,870,103,568 | 127,426,768 | 51.85 | 94.71 |
| L_2_-28 (P2) | 12,429,062,828 | 123,060,028 | 51.94 | 94.64 |
| L_4_-7 (F1) | 11,453,464,842 | 113,400,642 | 51.86 | 94.62 |
| L_2_-22 (P´1) | 13,516,387,014 | 133,825,614 | 51.75 | 94.82 |
| L_2_-20 (P´2) | 12,359,879,040 | 122,375,040 | 51.38 | 94.84 |
| L_4_-104 (F´1) | 12,511,565,688 | 123,876,888 | 51.66 | 94.76 |
| Total |  | 743,964,980 |  |  |
| **Trimmed data** | | | | |
| L_2_-4 (P1) | 11,904,610,008 | 121,866,682 | 51.47 | 98.58 |
| L_2_-28 (P2) | 11,490,241,014 | 117,706,278 | 51.54 | 98.55 |
| L_4_-7 (F1) | 10,578,943,407 | 108,384,826 | 51.46 | 98.57 |
| L_2_-22 (P´1) | 12,530,030,581 | 128,284,718 | 51.39 | 98.57 |
| L_2_-20 (P´2) | 11,455,707,993 | 117,179,384 | 51.00 | 98.60 |
| L_4_-104 (F´1) | 11,583,123,761 | 118,526,856 | 51.28 | 98.59 |
| Total |  | 711,948,744 |  |  |

Q20: The probability of an incorrect base call is ≤1%
