# Supplementary material for: Transcriptome Analysis by RNA–Seq Reveals Genes Related to Plant Height in Two Sets of Parent-hybrid Combinations in Easter lily (Lilium longiflorum)
Source: Sci Rep. 2020 Jun 3;10:9082. doi: 10.1038/s41598-020-65909-x (PMC7270119; doi:10.1038/s41598-020-65909-x)
Supplement: Supplementary file 1 — Supplementary information. [file 41598_2020_65909_MOESM1_ESM.zip › Supplementary files/Table S7.docx]

Table S7. The 59 most enriched KEGG pathway terms among the 260 functional transcripts sequences in *L. longiflorum*

| SL. NO. | Types of pathway | | In pathway | | | |
| --- | --- | --- | --- | --- | --- | --- |
|  |  |  | DEGs sequences | | Enzyme | |
|  | Name | ko-term | Number | ID | Name | ID |
| 1 | Biosynthesis of antibiotics | ko01130 | 8 | c34621_g1_i1 | monooxygenase | ec:1.14.13.72 |
|  |  |  |  | c15029_g1_i1 | synthase | ec:2.2.1.6 |
|  |  |  |  | c49546_g3_i1, c49546_g1_i1, c49546_g2_i1, c90374_g1_i1 | aldolase | ec:4.1.2.13 |
|  |  |  |  | c40971_g1_i1 | dehydrogenase | ec:1.1.1.37 |
|  |  |  |  | c55462_g1_i1 | adenylyltransferase | ec:2.7.7.4 |
| 2 | Phenylpropanoid biosynthesis | ko00940 | 6 | c59250_g1_i1 | gentiobiase | ec:3.2.1.21 |
|  |  |  |  | c57236_g2_i1, c2686_g1_i1, c55363_g3_i1, c55363_g3_i2, c62476_g1_i1 | lactoperoxidase | ec:1.11.1.7 |
| 3 | Pyrimidine metabolism | ko00240 | 5 | c53956_g1_i2, c53956_g1_i4, c43368_g1_i1, c53956_g2_i1 | synthase (glutamine hydrolysing) | ec:6.3.4.2 |
|  |  |  |  | c48799g1i2 | DNA polymerase | ec:2.7.7.7 |
| 4 | Purine metabolism | ko00230 | 5 | c47423_g1_i1, c54944_g1_i1, c43488_g1_i3 | phosphatase | ec:3.6.1.15 |
|  |  |  |  | c47423_g1_i1, c54944_g1_i1, c43488_g1_i3 | adenylpyrophosphatase | ec:3.6.1.3 |
|  |  |  |  | c55462_g1_i1 | adenylyltransferase | ec:2.7.7.4 |
|  |  |  |  | c48799_g1_i2 | DNA polymerase | ec:2.7.7.7 |
|  |  |  |  | c55462_g1_i1 | kinase | ec:2.7.1.25 |
| 5 | Carbon fixation in photosynthetic organisms | ko00710 | 5 | c49546_g3_i1, c49546_g1_i1, c49546_g2_i1, c90374_g1_i1 | aldolase | ec:4.1.2.13 |
|  |  |  |  | c40971_g1_i1 | dehydrogenase | ec:1.1.1.37 |
| 6 | Methane metabolism | ko00680 | 5 | c49546_g3_i1, c49546_g1_i1, c49546_g2_i1, c90374_g1_i1 | aldolase | ec:4.1.2.13 |
|  |  |  |  | c40971_g1_i1 | dehydrogenase | ec:1.1.1.37 |
| 7 | Fructose and mannose metabolism | ko00051 | 4 | c49546_g3_i1, c49546_g1_i1, c49546_g2_i1, c90374_g1_i1 | aldolase | ec:4.1.2.13 |
| 8 | Glycolysis / Gluconeogenesis | ko00010 | 4 | c49546_g3_i1, c49546_g1_i1, c49546_g2_i1, c90374_g1_i1 | aldolase | ec:4.1.2.13 |
| 9 | Pentose phosphate pathway | ko00030 | 4 | c49546_g3_i1, c49546_g1_i1, c49546_g2_i1, c90374_g1_i1 | aldolase | ec:4.1.2.13 |
| 10 | Thiamine metabolism | ko00730 | 3 | c47423_g1_i1, c54944_g1_i1, c43488_g1_i3 | phosphatase | ec:3.6.1.15 |
| 11 | Sulfur metabolism | ko00920 | 3 | c55462_g1_i1 | adenylyltransferase | ec:2.7.7.4 |
|  |  |  |  | c59180_g1_i1 | sulfurtransferase | ec:2.8.1.1 |
|  |  |  |  | c55462_g1_i1 | kinase | ec:2.7.1.25 |
| 12 | Starch and sucrose metabolism | ko00500 | 2 | c62272g2i1, | pectin depolymerase | ec:3.2.1.15 |
|  |  |  |  | c43045_g1_i1 |  |  |
| 13 | Drug metabolism - cytochrome P450 | ko00982 | 2 | c50413g1i1 | transferase | ec:2.5.1.18 |
|  |  |  |  | c63669_g1_i2 |  |  |
| 14 | Pentose and glucuronate interconversions | ko00040 | 2 | c62272g2i1, | pectin depolymerase | ec:3.2.1.15 |
|  |  |  |  | c43045_g1_i1 |  |  |
| 15 | Other glycan degradation | ko00511 | 2 | c33577g2i1 | lactase (ambiguous) | ec:3.2.1.23 |
|  |  |  |  | c62332g1i1 | alpha-fucosidase | ec:3.2.1.51 |
| 16 | Pyruvate metabolism | ko00620 | 1 | c40971_g1_i1 | dehydrogenase | ec:1.1.1.37 |
| 17 | Metabolism of xenobiotics by cytochrome P450 | ko00980 | 1 | c63669g1i2 | transferase | ec:2.5.1.18 |
| 18 | Galactose metabolism | ko00052 | 1 | c57944_g1_i1 | invertase | ec:3.2.1.26 |
| 19 | Glycine, serine and threonine metabolism | ko00260 | 1 | c56667g1i2 | kinase | ec:2.7.1.39 |
| 20 | Steroid degradation | ko00984 | 1 | c63435g1i2 | 4-dehydrogenase (acceptor) | ec:1.3.99.5 |
| 21 | Citrate cycle (TCA cycle) | ko00020 | 1 | c40971_g1_i1 | dehydrogenase | ec:1.1.1.37 |
| 22 | Flavone and flavonol biosynthesis | ko00944 | 1 | c56073g1i1 | 3'-O-methyltransferase | ec:2.1.1.42 |
| 23 | Glycosaminoglycan degradation | ko00531 | 1 | c33577g2i1 | lactase (ambiguous) | ec:3.2.1.23 |
| 24 | Aminobenzoate degradation | ko00627 | 1 | c43735_g1_i1 | nitrophenyl phosphatase | ec:3.1.3.41 |
| 25 | Limonene and pinene degradation | ko00903 | 1 | c62938g1i1 | 6-monooxygenase | ec:1.14.13.48 |
| 26 | Monoterpenoid biosynthesis | ko00902 | 1 | c62938g1i1 | 6-monooxygenase | ec:1.14.13.48 |
| 27 | Glutathione metabolism | ko00480 | 1 | c63669g1i2 | transferase | ec:2.5.1.18 |
| 28 | Glycosphingolipid biosynthesis - ganglio series | ko00604 | 1 | c33577g2i1 | lactase (ambiguous) | ec:3.2.1.23 |
| 29 | Sphingolipid metabolism | ko00600 | 1 | c33577g2i1 | lactase (ambiguous) | ec:3.2.1.23 |
| 30 | Glyoxylate and dicarboxylate metabolism | ko00630 | 1 | c56155g1i1 | (Si)-synthase | ec:2.3.3.1 |
| 31 | Steroid biosynthesis | ko00100 | 1 | c34621_g1_i1 | monooxygenase | ec:1.14.13.72 |
| 32 | Linoleic acid metabolism | ko00591 | 1 | c59526_g2_i1 | 13S-lipoxygenase | ec:1.13.11.12 |
| 33 | Nitrogen metabolism | ko00910 | 1 | c27330_g1_i1 | dehydrogenase [NAD(P)+] | ec:1.4.1.3 |
|  |  |  |  | c27330_g1_i1 | dehydrogenase | ec:1.4.1.2 |
| 34 | Carotenoid biosynthesis | ko00906 | 1 | c61841_g2_i1 | desaturase | ec:1.3.5.6 |
| 35 | Cysteine and methionine metabolism | ko00270 | 1 | c40971_g1_i1 | dehydrogenase | ec:1.1.1.37 |
| 36 | Porphyrin and chlorophyll metabolism | ko00860 | 1 | c64060_g3_i1 | reductase | ec:1.3.1.33 |
| 37 | Aminoacyl-tRNA biosynthesis | ko00970 | 1 | c63055_g2_i2 | ligase | ec:6.1.1.18 |
| 38 | Pantothenate and CoA biosynthesis | ko00770 | 1 | c15029_g1_i1 | synthase | ec:2.2.1.6 |
| 39 | Butanoate metabolism | ko00650 | 1 | c15029_g1_i1 | synthase | ec:2.2.1.6 |
| 40 | Ubiquinone and other terpenoid-quinone biosynthesis | ko00130 | 1 | c61288_g1_i1 | synthase | ec:4.1.3.36 |
| 41 | Selenocompound metabolism | ko00450 | 1 | c55462_g1_i1 | adenylyltransferase | ec:2.7.7.4 |
| 42 | Alanine, aspartate and glutamate metabolism | ko00250 | 1 | c27330_g1_i1 | dehydrogenase [NAD(P)+] | ec:1.4.1.3 |
|  |  |  |  | c27330_g1_i1 | dehydrogenase | ec:1.4.1.2 |
| 43 | Glycosaminoglycan biosynthesis - heparan sulfate / heparin | ko00534 | 1 | c63972_g2_i2 | 3-beta-galactosyltransferase | ec:2.4.1.134 |
| 44 | Glycosaminoglycan biosynthesis - chondroitin sulfate /dermatan sulfate | ko00532 | 1 | c63972_g2_i2 | 3-beta-galactosyltransferase | ec:2.4.1.134 |
| 45 | Valine, leucine and isoleucine biosynthesis | ko00290 | 1 | c15029_g1_i1 | synthase | ec:2.2.1.6 |
| 46 | Glycerophospholipid metabolism | ko00564 | 1 | c43735_g1_i1 | phosphatase | ec:3.1.3.75 |
| 47 | Amino sugar and nucleotide sugar metabolism | ko00520 | 1 | c57541_g1_i1 | chitodextrinase | ec:3.2.1.14 |
| 48 | Carbon fixation pathways in prokaryotes | ko00720 | 1 | c40971_g1_i1 | dehydrogenase | ec:1.1.1.37 |
| 49 | Vitamin B6 metabolism | ko00750 | 1 | c43735_g1_i1 | phosphatase | ec:3.1.3.74 |
| 50 | Glyoxylate and dicarboxylate metabolism | ko00630 | 1 | c40971_g1_i1 | dehydrogenase | ec:1.1.1.37 |
| 51 | Taurine and hypotaurine metabolism | ko00430 | 1 | c27330_g1_i1 | dehydrogenase | ec:1.4.1.2 |
| 52 | alpha-Linolenic acid metabolism | ko00592 | 1 | c59526_g2_i1 | 13S-lipoxygenase | ec:1.13.11.12 |
| 53 | D-Glutamine and D-glutamate metabolism | ko00471 | 1 | c27330_g1_i1 | dehydrogenase [NAD(P)+] | ec:1.4.1.3 |
| 54 | Arachidonic acid metabolism | ko00590 | 1 | c51727_g1_i2 | epoxide hydrolase | ec:3.3.2.10 |
| 55 | C5-Branched dibasic acid metabolism | ko00660 | 1 | c15029_g1_i1 | synthase | ec:2.2.1.6 |
| 56 | Arginine biosynthesis | ko00220 | 1 | c27330_g1_i1 | dehydrogenase [NAD(P)+] | ec:1.4.1.3 |
|  |  |  | 1 | c27330_g1_i1 | dehydrogenase | ec:1.4.1.2 |
| 57 | Cyanoamino acid metabolism | ko00460 | 1 | c59250_g1_i1 | gentiobiase | ec:3.2.1.21 |
| 58 | Monobactam biosynthesis | ko00261 | 1 | c55462_g1_i1 | adenylyltransferase | ec:2.7.7.4 |
| 59 | Chloroalkane and chloroalkene degradation | ko00625 | 1 | c51727_g1_i2 | epoxide hydrolase | ec:3.3.2.10 |
