# Supplementary material for: Transcriptome Analysis by RNA–Seq Reveals Genes Related to Plant Height in Two Sets of Parent-hybrid Combinations in Easter lily (Lilium longiflorum)
Source: Sci Rep. 2020 Jun 3;10:9082. doi: 10.1038/s41598-020-65909-x (PMC7270119; doi:10.1038/s41598-020-65909-x)
Supplement: Supplementary file 1 — Supplementary information. [file 41598_2020_65909_MOESM1_ESM.zip › Supplementary files/Title page.docx]

**Transcriptome Analysis by RNA–Seq Reveals Genes Related to Plant Height in Two Sets of Parent-hybrid Combinations in Easter lily** (***Lilium longiflorum)***

Jewel Howlader^1,2^, Arif Hasan Khan Robin^1,3^, Sathishkumar Natarajan^1^, Manosh Kumar Biswas^1^, Kanij Rukshana Sumi^4,5^, Cheon Young Song^6^, Jong–In Park^1^, Ill–Sup Nou^1*^

**Supplementary materials**

**Table S1**. Primer design for quantitative real–time PCR (qPCR) of commonly expressed DEGs from RNA sequencing data in *L*. *longiflorum*

**Table S2**. Quality of RNA sequencing data

**Table S3**. Summery details of unigenes in *L*. *longiflorum*

**Table S4A**. Estimations of commonly expressed DEGs in hybrids from parents

**Table S4B**. Fold change (FC) values of commonly expressed transcripts in hybrids compared to parents.

**Table S4C**. Sequence of commonly expressed DEGs in *L*. *longiflorum*

**Table S5A**. Functional characterization of commonly expressed up– and down–regulated DEGs in two sets of lily cross combinations using FPKM values

**Table S5B**. Functional characterization of commonly expressed up– and down–regulated DEGs in two sets of lily cross combinations using FPKM values

**Table S6**. Distribution of DEGs into different categories of Gene Ontology (GO) in *L*. *longiflorum*

**Table S7**. The 59 most enriched KEGG pathway terms among the 260 functional transcripts sequences in *L. longiflorum*

**Figure S1**. Correlation co–efficient (R2) between gene expression data sets from biological replicates of two hybrids (L4–7b) and (L4–104b), and the four parents (L2–4), (L2–28), (L2–22), and (L2–20)

**Figure S2**. Scatterplots showed transcripts expression levels as fold change values (FC) between hybrids L4–7 (F1) and L4–104 (F´1), and their respective four parents L2–4 (P1), L2–28 (P2), L2–22 (P´1), and L2–20 (P´2). A, L4–7 (F1)_versus_ L2–4 (P1), B, L4–7 (F1)_versus_ L2–28 (P2), C, L4–104 (F´1)_versus_ L2–22 (P´1), and D, L4–104 (F´1)_versus_ L2–20 (P´2). Here, red and gray colored dots denote significant (FC >= 2) and non–significant (FC < 2) transcripts expression levels, respectively.

**Figure S3**. Distribution of commonly expressed 703 DEGs showing functional annotation.
